# Massive Acromioclavicular Joint Cyst with Intramuscular Extension: Case Report and Review

**DOI:** 10.1155/2018/7602549

**Published:** 2018-06-10

**Authors:** Yiyang Zhang, Jason Old

**Affiliations:** ^1^Section of Orthopaedic Surgery, Department of Surgery, Rady College of Medicine, University of Manitoba, Winnipeg, MB, Canada; ^2^Pan Am Clinic, Winnipeg, MB, Canada

## Abstract

Acromioclavicular cysts are an uncommon manifestation secondary to a massive rotator cuff tear and/or a degenerative osteoarthritic AC joint. We present a case of an 80-year-old female with a symptomatic acromioclavicular cyst that extended intramuscularly into the trapezius. She did not complain of symptoms associated with a massive rotator cuff tear; however, the cyst has been increasing in size and she was interested in having it removed. Intraoperatively, the mass extended into the trapezius muscle and was removed en bloc after dissecting it down to the stalk. A distal clavicle excision was then performed using an oscillating saw. After the cyst was excised, it was incised revealing thick mucoid content. The patient did well postoperatively at the three-month follow-up without signs of recurrence. To our knowledge, this is the first case of AC joint cyst with intramuscular extension that was managed operatively.

## 1. Introduction

Acromioclavicular (AC) joint cysts are an uncommon sequela of full-thickness rotator cuff tears and degenerative AC joint changes. Composed of thick mucoid content enveloped by a fibrous wall, they usually present as a mass over the AC joint, which may raise concerns of tumor formation. An arthrogram may demonstrate fluid erupting superiorly through the AC joint, hence the term “geyser sign”. When indicated, surgical management of AC joint cysts should address the underlying pathology of rotator cuff tear in addition to cyst removal to avoid recurrence.

## 2. Case Presentation

An 80-year-old female presented to clinic with a mass over the superior aspect of the right scapula. The mass was achy but did not interfere with performing activities of daily living. However, it was bothersome for the patient and she stated that it had been enlarging over the previous few months. She denies any local injuries or recent surgeries on the affected side. She denied having weakness in the left arm when compared to the contralateral side. She denied having trouble with overhead activities. She had not noticed any constitutional symptoms or nighttime pain. She did give a history of having a similar mass on the contralateral side, which was excised 10 years previously with a favorable result. She was very interested in having the new mass excised as well.

Inspection of the area is unremarkable, but palpation of the area demonstrates a firm, nonmobile, and nonpulsatile mass in the area of the upper trapezius overlying the scapula. The mass is longer in its medial to lateral dimension than craniocaudad. With deeper palpation, slight tenderness can be elicited. Examination of the shoulder does not yield signs of rotator cuff weakness or shoulder pain with provocative maneuvers.

Plain X-ray demonstrates narrowing of the posterior glenohumeral joint space with sclerosis secondary to osteoarthritic changes. The acromiohumeral interval is measured to be 8 mm without signs of superior migration of the humeral head.

**Figure 1:**
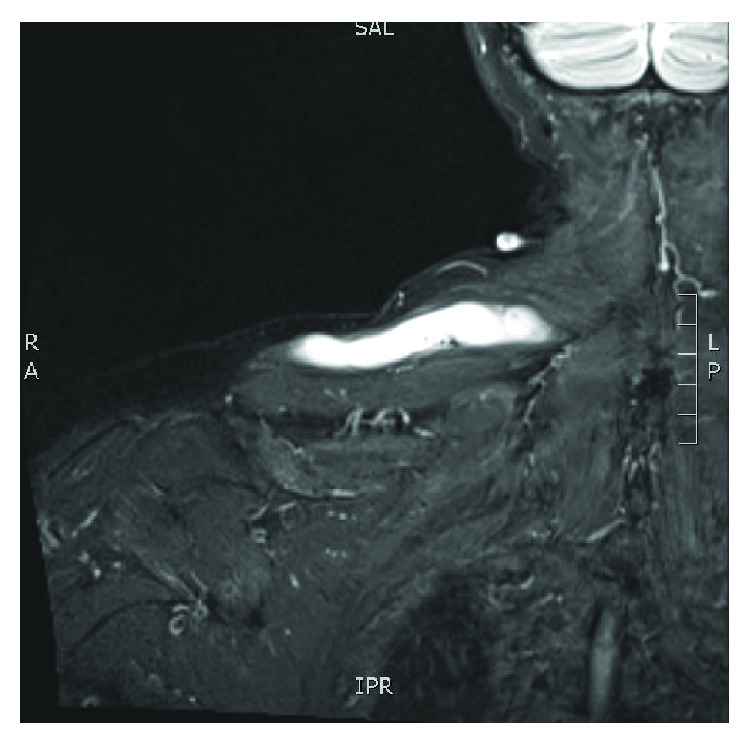
Coronal cuts of MRI of the right thorax depicting cyst measuring 2 cm (AP) × 13 (transverse) × 1.8 cm (craniocaudad).

**Figure 2:**
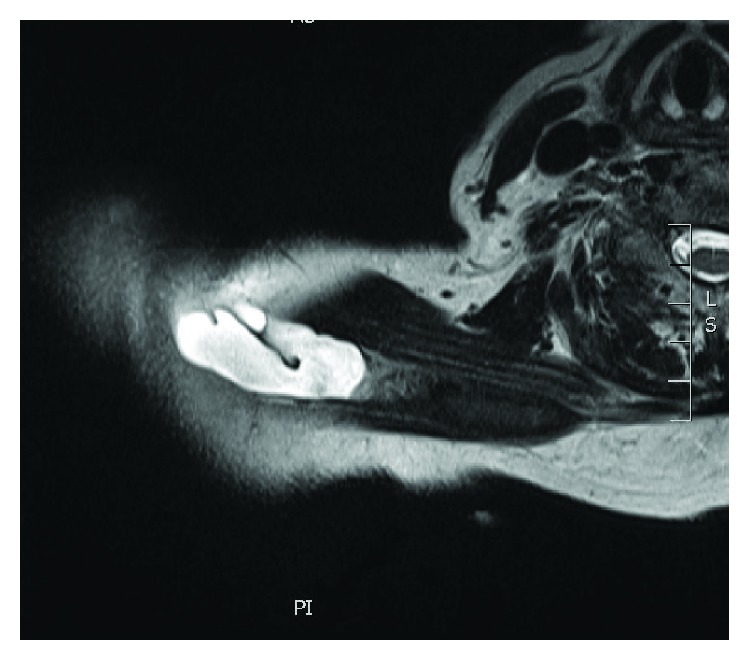
Axial cuts of MRI of the right thorax depicting cyst measuring 2 cm (AP) × 13 (transverse) × 1.8 cm (craniocaudad).

Magnetic resonance imaging (MRI) shows an elongated lesion arising from the AC joint and tracking medially to superficial and within the trapezius muscle (Figures 1 and 2). It measures 2 cm (AP) × 13 cm (transverse) × 1.8 cm (craniocaudad). The lesion appears cystic with peripheral enhancement. There is also suspected full-thickness tear of the anterior fibers of the supraspinatus.

**Figure 3:**
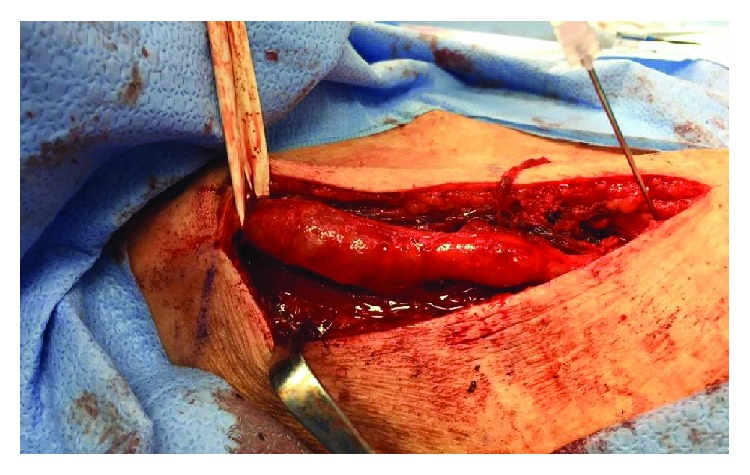
Intraoperative identification of cyst tracking from the AC joint (spinal needle) medially.

**Figure 4:**
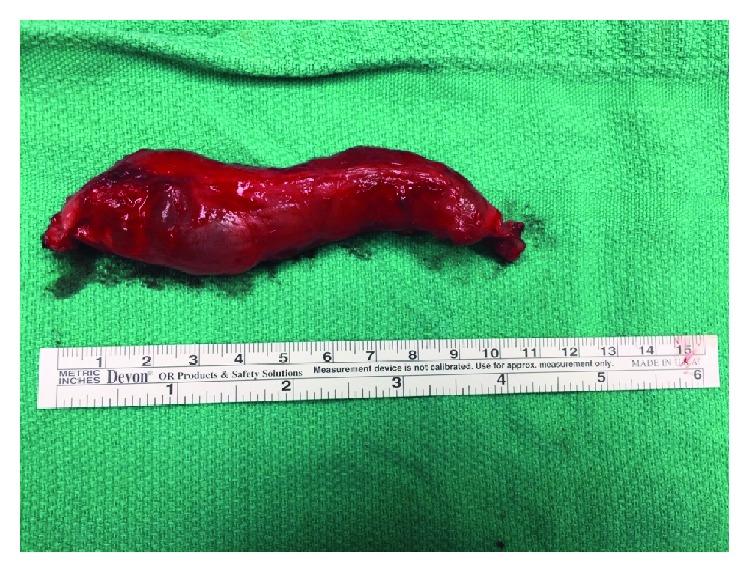
Cyst removed en bloc.

**Figure 5:**
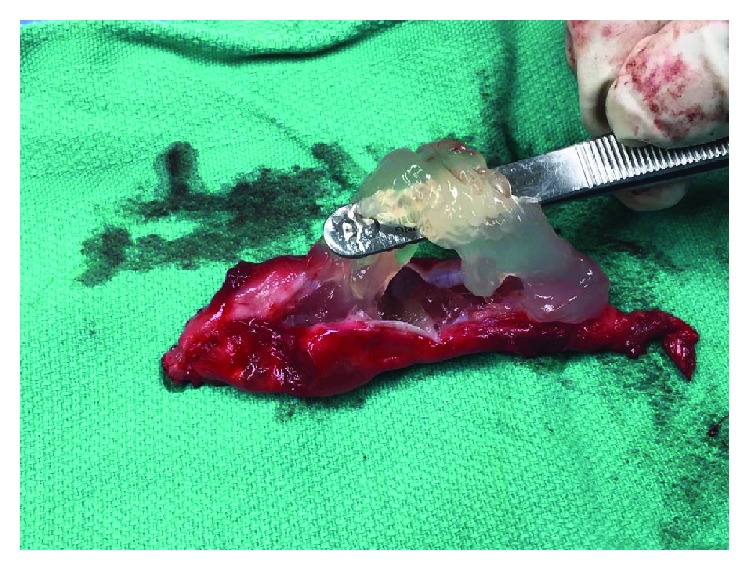
Incising the cyst open revealed thick mucoid content.

Although the mass did not prevent the patient from performing her activities of daily living, it was bothersome enough for her that she wanted it removed. Under a general anesthetic, an incision was made directly over the palpable mass. With careful dissection, a stalk emanating from the AC joint was identified. The mass extended laterally from the AC joint within the trapezius muscle for approximately 13 cm (Figure 3). Once it was dissected free, the mass was removed en bloc. A distal clavicle excision was then performed using an oscillating saw. After the cyst was excised, it was incised revealing thick mucoid content (Figures 4 and 5).

The patient was followed up at three weeks and three months postoperatively. She said she felt pain relief immediately after the surgery and she continued to have full, pain-free range of motion with no signs of recurrence.

## 3. Discussion

Acromioclavicular joint cysts are a rare clinical entity. They are thought to occur secondary to chronic rotator cuff tears with AC joint arthritis. The pathogenesis of the cyst is thought to relate to increased fluid from the rotator cuff tear escaping through a defect in the AC joint capsule which acts as a one-way valve.

AC joint cysts present as a mass over the AC joint which is often painless. While aspiration is often performed, it is generally discouraged due to a high recurrence rate and risk of formation of fistula [1, 2]. Operative treatment of AC joint cysts is generally reserved for cases where there is significant pain, or when the cyst is large and aesthetically unacceptable to the patient.

To our knowledge, this is only the second case of intramuscular extension of the cyst medially within the trapezius muscle and the first to be managed operatively. Montet et al. described an AC joint cyst located inside the trapezius that was confirmed with ultrasound and MRI [3]. That patient went on to be managed nonoperatively given her general lack of symptoms. Sanders et al. report a series of 13 ganglia located inside rotator cuff muscles when they performed MRI on 1150 shoulders [4]. The more common area where ganglionic cysts dissect through muscle is in the anterolateral compartment of the leg, which can lead to peroneal nerve compression secondary to mass effect [4, 5].

When AC joint cysts were first described by Craig in 1976, arthrogram was the test of choice. The spewing of fluid from the AC joint was termed the “geyser sign” [6, 7]. MRI is now the imaging modality of choice given its wide availability and noninvasiveness. On T1, AC joint cysts demonstrate decreased signal intensity and increased signal intensity on T2-weighted sequences.

Hiller et al. described two types of AC joint cysts. Type 1 is characterized with no communication with the glenohumeral joint, while type 2 is defined by a rotator cuff tear which allows for a fluid channel to be established between the AC and GH joints [8]. An increase in production of intra-articular synovial fluid and superior migration of humeral head secondary to the rotator cuff tear can erode the inferior capsule of the AC joint allowing fluid to pass through and cause distention. Separating the two pathologies can help establish treatment options.

Both type 1 and 2 AC joint cysts can be managed nonoperatively or operatively depending on the patient's symptoms, age, medical comorbidities, and the presence of a rotator cuff tear. Type 1 cysts, given the lack of communication with the GH joint, can be treated with distal clavicle excision and subacromial bursectomy [9]. Type 2 cysts often present with chronic massive rotator cuff tear that may not be able to be addressed directly given its chronicity and extent. Feeley et al. proposed treatment options of arthroscopy and debridement, hemiarthroplasty, total shoulder arthroplasty, reverse total shoulder arthroplasty, and shoulder arthrodesis [10].

In our patient, we removed the AC cysts in its entirety and performed a distal clavicle excision to alleviate the irritation on the synovium caused by the degenerative changes of the AC joint. Given our patient's overall rotator cuff strength and lack of pain on provocative maneuvers on physical exam, we decided not to address the suspected full-thickness rotator cuff tear. Presently, the patient is asymptomatic with no signs of recurrence.

## 4. Conclusion

Acromioclavicular joint cysts are an uncommon presentation of AC joint degeneration and rotator cuff tear or arthropathy. It usually presents as a relatively painless lump directly over the AC joint. Our patient's AC joint cyst tracked medially along and through the trapezius muscle. To our knowledge, we are the first to manage this type of lesion surgically. With removing the cyst en bloc and performing a distal clavicle excision, the patient is so far satisfied with her outcome without signs of recurrence. Longer-term follow-up and report of more cases can help with confirmation of the effectiveness of this surgical approach.
